# Multimorbidity and Everyday Discrimination Among Young, Middle-Aged, and Older Black Adults

**DOI:** 10.1093/geroni/igaf122.1688

**Published:** 2025-12-31

**Authors:** Courtney Thomas Tobin, Heather Farmer, Angela Gutierrez, Xumeng Yan, Hans Oh, Roland Thorpe

**Affiliations:** University of California, Los Angeles Fielding School of Public Health, Los Angeles, California, United States; University of Delaware, Newark, Delaware, United States; Western University of Health Sciences, Pomona, California, United States; University of California, Los Angeles Fielding School of Public Health, Los Angeles, California, United States; University of Southern California, Los Angeles, California, United States; Johns Hopkins Bloomberg School of Public Health, Baltimore, Maryland, United States

## Abstract

Multimorbidity, the presence of two or more chronic conditions, disproportionately affects Black Americans and contributes to racial disparities in disability and mortality. Everyday discrimination is a chronic psychosocial stressor associated with multimorbidity. Although prior research suggests chronic stress accumulates and manifests differently over time, it remains unclear whether the association between discrimination and multimorbidity varies across adult life course age groups. Using data from the Nashville Stress and Health Study (n = 627), this study examined the association between everyday discrimination and multimorbidity and whether it varied significantly among young (ages 18-35), middle-aged (ages 36-49), and older (ages 50+) Black Americans. Multimorbidity was based on the sum of 11 chronic physical health conditions, and we used the Everyday Discrimination Scale to assess discrimination exposure. We estimated negative binomial regression models with an interaction term between discrimination and age. Results showed a greater likelihood of multimorbidity among older adults (IRR=3.21, 95% CI = 2.64-3.86, p > 0.001) and a positive association with everyday discrimination (IRR = 1.03, 95% CI = 1.01-1.05, p < .05). A significant interaction term (F(2, 256) = 13.75, p < 0.001) indicated the discrimination-multimorbidity association was strongest among young adults and decreased incrementally among middle-aged and older adults. This suggests discrimination may accelerate multimorbidity among younger adults, whereas its impact diminishes in later life as other factors impact the aging process. These findings underscore the need for early-life interventions to mitigate the health consequences of discrimination and prevent the early onset of multimorbidity among Black Americans.

